# Boosting the inherent activity of NiFe layered double hydroxide via erbium incorporation for water oxidation

**DOI:** 10.3389/fchem.2023.1261332

**Published:** 2023-08-24

**Authors:** Jitao Yang, Yibin Yang

**Affiliations:** School of Chemical and Pharmaceutical Engineering, Chongqing Industry Polytechnic College, Chongqing, China

**Keywords:** NiFe layered double hydroxide, oxygen evolution reaction, erbium incorporation, electrocatalysis, inherent activity

## Abstract

Enhancing the inherent activity of transition metal-based compounds involving Ni and Fe for the electrocatalytic oxygen evolution reaction (OER) is of vital importance, especially NiFe layered double hydroxide (LDH). Here, we doped erbium (Er) into NiFe LDH (Er–NiFe LDH) nanostructures using simple liquid-phase synthesis. The OER activity tests at the same mass loading demonstrated that Er–NiFe LDH has a smaller overpotential and lower Tafel slope than undoped NiFe LDH and commercial RuO_2_ powders, needing only a small overpotential of 243 mV to achieve a constant current at 10 mA cm^-2^. Additionally, Er–NiFe LDH was grown *in situ* on hydrophilic carbon paper substrates (Er–NiFe LDH@CP) to fabricate a three-dimensional (3D) electrode with large catalyst loading, which is favorable for analyzing the stability of morphology structure and elementary components after OER measurement. The galvanostatic measurement suggested that the Er–NiFe LDH@CP electrode possess higher electrochemical durability than a modified glassy carbon electrode due to the stronger mechanical binding between Er–NiFe LDH nanostructures and carbon paper substrate. More importantly, physical characterizations (e.g., SEM and XPS) revealed that Er–NiFe LDH has an excellent stability of morphology, and Ni, Fe, and Er still exist on the catalyst 24 h after the operation. This work provides an effective way for improving the inherent catalytic activity and stability of polymetallic OER catalysts in the future.

## 1 Introduction

Hydrogen is an alternative energy source with high efficiency and is pollution-free compared with non-renewable energy sources like petroleum, coal, natural gas, and geothermal energy (Lewis and Nocera, 2006; Yang X. et al., 2022). Alkaline water electrolysis is an important technology for the production of green hydrogen (Lagadec and Grimaud, 2020; Yang et al., 2022b). However, the anodic oxygen evolution reaction (OER) with slow kinetic and high overpotential severely hinders the industrial application of water decomposition technology (Zhao et al., 2023). This has brought to the necessity to exploit an efficient electrocatalyst that can accelerate the OER rate and lower the overpotential (Roger et al., 2017). Currently, precious metal catalysts, such as IrO_2_ and RuO_2_, exhibit considerable catalytic performance, but their wide usage was limited due to their high production cost (Lee et al., 2012; Sun et al., 2015). Therefore, many researchers are devoted to developing earth-abundant catalysts much further.

NiFe layered double hydroxide (LDH) has recently received remarkable attention in both academia and industry by virtue of its sufficient reserves, flexible and tunable composition, and good inherent activity (Trotochaud et al., 2014; Yang et al., 2018; Guo et al., 2021; Yang et al., 2023). However, further enhancing the inherent activity of NiFe LDH for water oxidation remains a major challenge. Many approaches have been used to improve their inherent activity (Liang et al., 2015; Zhou et al., 2021). For instance, Song and Hu (2014) and Wang et al. (2017) prepared monolayer LDH nanosheets by liquid-phase and dry exfoliations, respectively. The exfoliated LDH exhibits a higher activity than bulk LDH materials, which is primarily attributed to more active sites and improved conductivity and created multiple vacancies of the former. Our prior works demonstrate that doping Cr (Yang et al., 2018), W (Guo et al., 2021), and Ru (Yang et al., 2022c) cations are favorable for modulating the electronic interaction among three metal cations and enhancing the electronic conductivity of NiFe LDH, thereby improving their catalytic performance. Jin and coworkers tried to probe the relationship between interlayer spacing and OER performance (Dang et al., 2018). They prepared NiFe LDH with various interlaminate anions (e.g., SO_4_^2-^, Cl, and CO_3_^2-^) via anion exchange and direct synthesis under room atmosphere. Electrochemical measurements indicate that the intercalation of different anions does not influence the catalytic activity of NiFe LDH after the normalization of the electrochemical surface area. Although various aforementioned strategies have been attempted to enhance the OER performance of pure NiFe LDH, the improvement is still limited. The lanthanide-like erbium ion (Er^3+^) exhibits a 4f electronic configuration, which helps enhance conductivity and modulate the energy-level structure to promote the catalytic performance toward OER (Hao et al., 2020). Therefore, incorporation of erbium may be a useful way to boost the inherent activity of NiFe LDH toward electrocatalytic water oxidation.

Here, we doped trivalent erbium into the lattice of NiFe LDH to fabricate a highly active Er-incorporated NiFe LDH (labeled as Er–NiFe LDH) catalyst for catalyzing water oxidation using a one-step solvothermal reaction. The Er–NiFe LDH powder requires an overpotential of only 243 mV to reach 10 mA cm^-2^, and a Tafel slope of 77.1 mV decade^−1^ shows the higher inherent activity for the alkaline OER than that of undoped NiFe LDH. Moreover, to further promote the mechanical stability of the electrode containing Er–NiFe LDH, Er–NiFe LDH nanostructures were grown *in situ* on the carbon paper substrate (Er–NiFe LDH@CP) to develop a three-dimensional (3D) electrode, which shows excellent electrochemical durability in basic solution. Significantly, the morphology and elementary composition of the integrated electrode show no changes after long-term galvanostatic operation, according to the microscopy SEM and XPS analysis. This study provides a useful way for enhancing the catalytic performance and stability of NiFe LDH for water oxidation.

## 2 Materials and methods

### 2.1 Chemicals and materials

Nickel (II) nitrate hexahydrate (Ni(NO_3_)_2_·6H_2_O; 98%, CAS No. 7791-20-0, Alfa), iron (II) chloride tetrahydrate (FeCl_2_·4H_2_O; 99.95%, CAS No. 13478-10-9, Macklin), erbium(III) chloride (ErCl_3_; 99.9%, CAS No. 10138-41-7, Aladdin), urea (CH_4_N_2_O; 99%, CAS No. 57-13-6, Aladdin), Nafion solution (5%, CAS No. 31175-20-9, Sigma-Aldrich), hydrochloric acid (HCl; 36%–38%, CAS No. 7647-01-0, Sinopharm Group Chemical Reagent Co., Ltd.), ruthenium (IV) oxide (RuO_2_; 99.9%, CAS No. 12036-10-1, Aladdin), and potassium hydroxide (KOH; 90%, CAS No. 1310-58-3, Macklin) were used directly without further purification. The ultrapure water (≥18.25 MΩ cm) used in this work was obtained through the Ulupure UPR-III-10T (Sichuan Ulupure Ultrapure Technology Co., Ltd.) system. Toray carbon paper (CP; TGP-H-060, 5% wet proofing) was purchased from Fuel Cell Earth.

### 2.2 Synthesis of materials

#### 2.2.1 Er-doped NiFe LDH powders

The Er–NiFe LDH powder was synthesized by a simple solvothermal process. Briefly, 0.058 g of ErCl_3_ was dissolved in 8 mL of HCl solution (0.05 M) to form precursor A at room temperature. A volume of 0.795 g of FeCl_2_·4H_2_O and 0.348 g of Ni(NO_3_)_2_·6H_2_O were dissolved in 32 mL of ultrapure water to form precursor B. Next, 0.480 g of urea was completely dissolved in the mixed solution containing precursors A and B. Finally, 15 mL of the prepared precursor solution was transferred to the reaction vessel and then warmed at 120°C for 6 h. After the reaction was completed, the reactor was cooled down to 25°C. The obtained wet powder was centrifuged and washed at least three times and dried overnight at 60°C. The undoped NiFe LDH powder was also synthesized by a similar process without the introduction of the Er source.

#### 2.2.2 Growing Er–NiFe LDH on carbon paper

The carbon paper substrate was hydrophilic-treated, as described in Yang et al. (2022d) and Yang et al. (2022e). The precursor solution was prepared according to the aforementioned preparation procedures. A volume of 15 mL of the precursor solution and the processed carbon paper (1 cm × 4 cm) were transferred to the autoclave and then warmed at 120°C for 6 h. After the reaction was completed, the autoclave was cooled down to 25°C, and the Er–NiFe LDH@CP electrode was washed three times with ethanol and water and, subsequently, dried with pure nitrogen flow.

### 2.3 Material characterization

Raman spectra were detected using a DXR (Thermo Fisher). The scanning electron microscopy (SEM) images were collected using a ZEISS Sigma 300 operated at a voltage of 10 kV. PXRD patterns were recorded using a D8 ADVANCE (Bruker) spectrometer fitted with a Cu Kα radiation source (*λ* = 1.5418 Å) in the 2*θ* range of 5°–70° at a scan rate of 5° min^-1^. Fourier-transform infrared (FT-IR) spectra were measured using a Vector 22 (Bruker) infrared spectrophotometer in the wavenumber ranging between 400 and 4,000 cm^-1^. High-resolution transmission electron microscopy (HRTEM), SAED, and HAADF-STEM were performed using a F200X. XPS tests were recorded using a Thermo Scientific K-Alpha equipped with Al Kα rays (hν=1486.6 eV) as the excitation source at an operating voltage of 12 kV.

### 2.4 Electrochemical characterization

*Preparation of the working electrode*: Briefly, 5 mg of the Er–NiFe LDH powder, 600 µL of ultrapure water, 400 µL of ethanol, and 20 µL of Nafion solution were mixed and ultrasonicated for 30 min to generate homogenous ink. Next, 5 µL of the ink was dripped down onto the polished glassy carbon electrode (GCE; catalyst loading of ∼0.13 mg cm^-2^) and dried under an infrared lamp. Er–NiFe LDH@CP served directly as the working electrode with a catalyst loading of 1.36 mg cm^-2^.

*OER performance measurement*: All OER measurements were made on a typical three-electrode configuration via an electrochemical workstation (CHI760E) connected to the rotating disk electrode (RDE, Pine Research Instrumentation) composed of a glassy carbon tip (GC, AFE5T050GC, 5 mm diameter). The Hg/HgO/1 M KOH electrode and Pt wire served as the reference and counter electrodes, respectively. High-purity oxygen (99.999%) was purged on the 1.0 M KOH electrolyte (pH ∼13.89). All potentials were changed to reversible hydrogen electrodes (RHEs) based on the following Nernst equation: E_RHE_ = E_Hg/HgO_ + 0.059 × pH + 0.098 V. For 1 M KOH solution, E_RHE_ = E_Hg/HgO_ + 0.918 V. The cyclic voltammetry (CV) curves were collected in the range of 0.2–0.8 V *vs.* Hg/HgO at a scan rate of 5 mV s^-1^ and compensated by the 95% *i*R correction (Supplementary Figure S4). ECSA was calculated through double-layer capacitance (C_dl_), which was evaluated via the CV method in the scan range of 0.2–0.3 V *vs.* Hg/HgO at different scan rates. EIS measurement was performed at a voltage of 0.55 V *vs.* Hg/HgO in the frequency range of 500–1 kHz. The measurements of constant current were calculated at 10 mA cm^-2^ for 8 and 24 h.

## 3 Results and discussion

**FIGURE 1:**
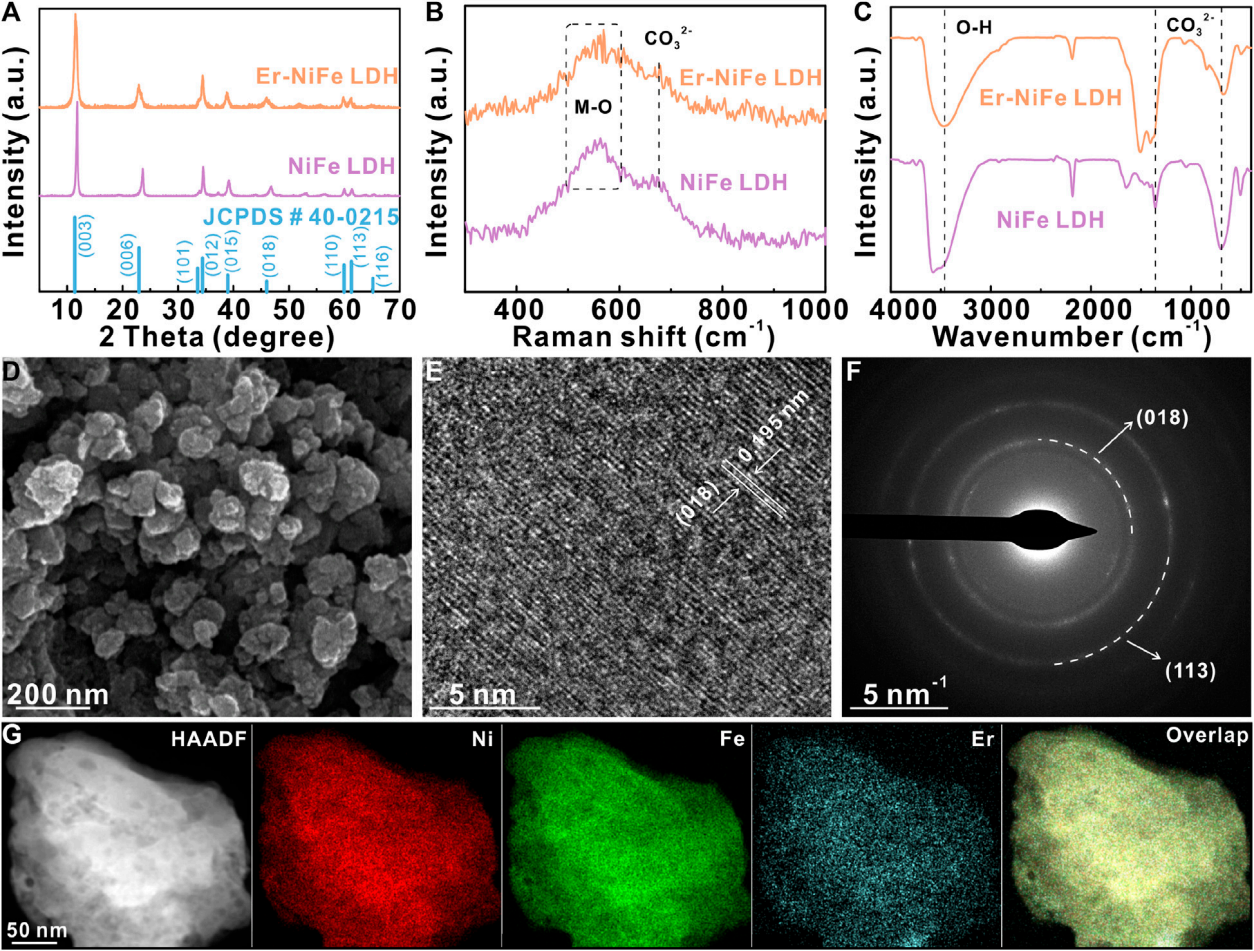
**(A)** PXRD patterns, **(B)** Raman spectra, and **(C)** FT-IR spectra of two LDH powders. **(D)** SEM image, **(E)** HRTEM image, **(F)** SAED pattern, and **(G)** HAADF–STEM image and corresponding elementary mappings of Er–NiFe LDH powders.

We prepared the Er–NiFe LDH powder by facile solvo/hydrothermal reactions, using HCl solution and ultrapure water as solvents and Ni(NO_3_)_2_·6H_2_O, ErCl_3_, and FeCl_2_·4H_2_O as the metal precursors. The PXRD pattern of Er–NiFe LDH (Figure 1A) shows a typical LDH structure and matches the reference NiFe LDH pattern well (JCPDS # 40-0215) (Yang et al., 2018; Guo et al., 2021; Yang et al., 2023). The enlarged PXRD pattern (Supplementary Figure S1) displays a slightly negative shift at 11.50° and 22.92° toward the lower angle after Er doping compared to undoped NiFe LDH. This indicates that doping with Er, which has larger atomic radius, triggers the lattice contraction and parameter decrease in crystals of NiFe LDH (Gao et al., 2019; Fan et al., 2020). Figure 1B displays the Raman spectra of two LDH samples, suggesting that the bands at 515–600 and 691 cm^-1^ correspond to the stretching vibration mode of M–O (M = Ni, Fe, and Er) and CO_3_^2-^, respectively (Kloprogge et al., 2002; Yang et al., 2023). FT-IR spectra were used to study the interlaminar anions of two LDH samples (Figure 1C). The peak at 3,469.69 cm^-1^ corresponds to hydrogen bonds in interlayered water molecules (Wang et al., 2018). The peaks at 1,406 and 676.96 cm^-1^ are assigned to carbonate ions (Pan et al., 2018). The results suggest that carbonate ions and H_2_O molecules are embedded in the interlayer of LDHs. Furthermore, Figures 1D and S2a show the morphology of nanoparticles and well-defined nanosheets of Er–NiFe LDH and undoped NiFe LDH samples, respectively. The results of the other physical characterization techniques (e.g., HRTEM, SAED, and HAADF-STEM) of the undoped NiFe LDH powder are shown in (Supplementary Figures S2C, D). The results reveal that Er doping can change the morphology of the NiFe LDH sample. The HRTEM image (Figure 1E) presents a sharp lattice stripe with an interplanar distance of 0.195 nm, which can be ascribed to the (018) crystal plane. The SAED pattern is further used to probe the crystal structure of the Er–NiFe LDH powder. As presented in Figure 1F, two groups of diffraction rings are ascribed to (018) and (113), respectively. This result is in accordance with two characteristic peaks at 46.0° and 61.2° in the PXRD pattern of the Er–NiFe LDH sample (Figure 1A). The HAADF-STEM image and corresponding elementary maps (Figure 1G) clearly illustrate that the metal elements of Ni, Er, and Fe are evenly scattered on the surface of Er–NiFe LDH powders.

**FIGURE 2:**
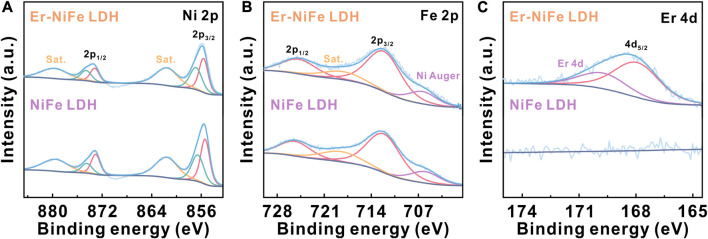
High-resolution XPS spectra for **(A)** Ni 2p, **(B)** Fe 2p, and **(C)** Er 4d of two LDH powders.

Furthermore, we performed XPS to explore the oxidation state and electron transfer of each element for two LDH samples. The survey XPS spectrum (Supplementary Figure S3A) suggests that Er–NiFe LDH consists of Ni, Er, O, Fe, and C elements. The high-resolution XPS spectra of Ni 2p and Fe 2p for two LDHs (Figures 2A,B) are divided into 2p_3/2_ and 2p_1/2_ peaks, respectively. The valence states of Ni and Fe in undoped NiFe LDH are +2 and +3 states, respectively (Li et al., 2018; Lin Y. P. et al., 2020). The Ni 2p_3/2_ peaks (Figure 2A) at 855.6 and 856.9 eV of Er–NiFe LDH correspond to Ni^2+^, which are in line with the reported results (Li et al., 2018). The Fe 2p_3/2_ peak at 712.1 eV corresponds to Fe^3+^, which indicates that Fe is trivalent (Lin Y. P. et al., 2020). It should be noted that the peak at 706.3 eV of Fe 2p spectra for two LDHs can be attributed to Ni Auger signals (Li et al., 2018; Lin Y. P. et al., 2020). Compared to Ni 2p and Fe 2p spectra of undoped NiFe LDH, the binding energy of Ni 2p_3/2_ shows a slight increase from 855.4 and 856.6 eV to 855.6 and 856.9 eV, while Fe 2p_3/2_ exhibits a minor decrease from 712.2 eV to 712.1 eV after erbium incorporation, suggesting that the incorporation of Er can regulate the electronic structure of undoped NiFe LDH. The high-resolution Er 4d spectra (Figure 2C) displays 4d_5/2_ (167.9 eV) and 4d (169.7 eV) peaks, which can be assigned to Er^3+^ (Qin et al., 2018; Hao et al., 2020). In addition, the C 1s spectra of all LDHs (Supplementary Figure S3B) show three fitted peaks located at 284.6, 285.7, and 289.0 eV, which correspond to C–C, O–C–O, and O–C=O, respectively (Yang et al., 2022c). Peak fitting of O 1s for two LDHs (Supplementary Figure S3C) demonstrates three peaks at 531.4, 530.1, and 532.4 eV, which can be attributed to M–O, M–OH, and H_2_O, respectively (Lin B. F. et al., 2020). These results illustrate that carbonate ions and water molecules are located in the interlayer of LDHs.

**FIGURE 3:**
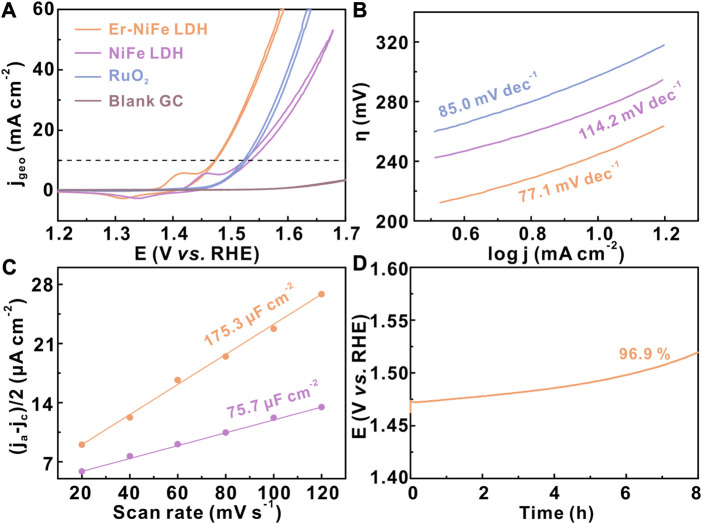
OER performance of two LDH samples in the 1 M KOH electrolyte. **(A)** CV curves, **(B)** Tafel plots, **(C)** C_dl_, and **(D)** electrochemical stability of Er–NiFe LDH via the constant current (10 mA cm^-2^) measurement. The catalyst loading was ∼0.13 mg cm^-2^.

**FIGURE 4:**
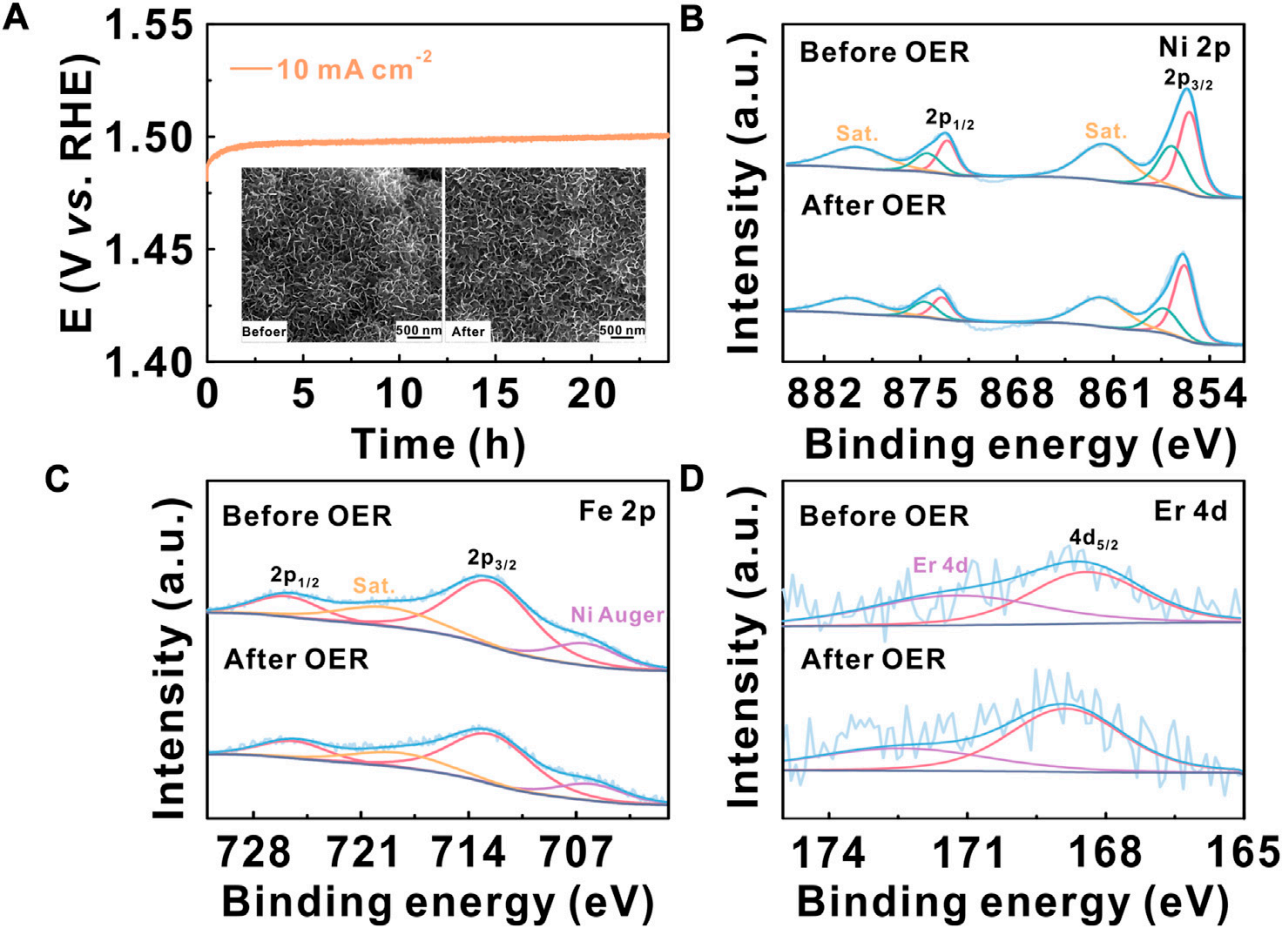
**(A)** Electrochemical stability of the Er–NiFe LDH@CP electrode at a constant current of 10 mA cm^-2^; the inset images show the SEM images before and after durability measurement. High-resolution XPS spectra for **(B)** Ni 2p, **(C)** Fe 2p, and **(D)** Er 4d of the Er–NiFe LDH@CP electrode before and after OER measurements.

To investigate the effect of the inherent activity of NiFe LDH after Er doping, the electrochemical test was performed on a normal three-electrode system connected to a potentiostat (CHI760E) and a rotating ring-disk electrode (RRDE) in the basic electrolyte. The modified glassy carbon electrodes with a similar catalyst loading (∼0.13 mg cm^-2^) are used as the working electrodes. All polarization curves are manually compensated by solution resistance (Supplementary Figure S4). Figure 3A shows the *i*R-compensated cyclic voltammetry curves of all samples, which shows that the Er–NiFe LDH sample displays excellent OER performance in the 1.0 M KOH electrolyte. Er–NiFe LDH requires only a small overpotential of 243 mV to reach 10 mA cm^-2^ compared with undoped NiFe LDH (294 mV) and commercial RuO_2_ (293 mV). The Tafel slope is also a key indicator for exploring OER kinetic (Stevens et al., 2017). Figure 3B indicates that Er–NiFe LDH has a lower Tafel slope of 77.1 mV dec^−1^ than NiFe LDH (114.2 mV dec^−1^) and RuO_2_ (85. mV dec^−1^), suggesting a faster reaction kinetic. Furthermore, to reveal how the catalytic activity is influenced by ECSA through double-layer capacitance (C_dl_) (Anantharaj et al., 2018), we tested C_dl_ of two LDH samples via the CV method (Figure 3C). The C_dl_ of Er–NiFe LDH (175.3 μF cm^-2^) is much higher than that of undoped NiFe LDH (75.7 μF cm^-2^), illustrating the presence of more active sites of Er–NiFe LDH. This suggests that the incorporation of Er is favorable for increasing the ECSA of undoped NiFe LDH. Electrochemical stability is another important parameter of electrocatalysts for water oxidation. The modified GC electrode deposited with Er–NiFe LDH was operated at a constant current test of 10 mA cm^-2^ for 8 h. Figure 4D demonstrates the good electrochemical stability of Er–NiFe LDH, and the overpotential shows only a slight decay after 8-h measurement. This is mainly due to the fragile combination between the Er–NiFe LDH powder and surface of the glassy carbon, although the Nafion solution served as a binder.

To further improve the electrochemical stability and probe the structural and composition stabilities of Er–NiFe LDH, Er–NiFe LDH nanostructures were directly grown on hydrophilic carbon paper substrates (Er-NiFe LDH@CP) to make a 3D electrode with a large catalyst loading for making it convenient for material characterization. Supplementary Figure S5 presents the SEM image, XRD pattern, and EDS elemental mappings of the Er–NiFe LDH@CP electrode, indicating the successful fabrication of the modified 3D electrode. The Er–NiFe LDH@CP electrode has approximately the same OER performance as that of Er–NiFe LDH powders on the GC electrode, which was confirmed based on the values of the overpotential and Tafel slope (Supplementary Figure S6). Figure 4A shows that the overpotential barely changes after 24-h operation, suggesting a better stability of the 3D electrode than the modified GC electrode. This can be attributed to the strong physical binding between Er–NiFe LDH nanostructures and the carbon paper substrate. The SEM images (inset of Figure 4A) show that the morphology of Er–NiFe LDH@CP remains constant before and after the OER measurement, demonstrating good structure stability. We also performed XPS to analyze the elementary compositions of the Er–NiFe LDH@CP electrode after OER measurement (Figures 4B–D and S7). After careful comparison, we found that the intensity and location of high-resolution Ni 2p, Fe 2p, and Er 4d XPS spectra showed no significant changes after evaluating stability. This reveals that Ni, Fe, and Er species still stably exist on the lattice of Er–NiFe LDH under harsh OER conditions, demonstrating the excellent composition stability of the Er–NiFe LDH electrocatalyst. This study introduces a useful avenue for the improvement of the inherent activity of NiFe LDH and the design of multi-metal-based electrocatalysts toward the basic OER in the future.

## 4 Conclusion

In conclusion, we have successfully incorporated Er into the lattice of NiFe LDH to promote their inherent activity toward OER using a simple hydrothermal reaction. The measurements of the OER activity with the same catalyst loading shows that Er–NiFe LDH has a lower overpotential at 10 mA cm^-2^ (243 mV) and a smaller Tafel slope (77.1 mV dec^−1^) than undoped NiFe LDH, which suggests that Er doping can really improve the intrinsic activity of NiFe LDH. Furthermore, Er–NiFe LDH nanostructures grown *in situ* on hydrophilic carbon paper substrates (Er-NiFe LDH@CP) exhibit stronger mechanical binding and more long-lasting electrochemical durability compared with the modified glassy carbon electrode after 24-h operation under harsh alkaline conditions. More importantly, physical characterizations (e.g., SEM and XPS) verify that the Er–NiFe LDH@CP electrode possesses an anti-collapse microstructure and elementary component. This work provides a practical and facile avenue to optimize the overall performance of NiFe LDH and fabricate more efficient NiFe-based catalysts toward electrocatalytic water oxidation for further research.

## Data Availability

The original contributions presented in the study are included in the article/Supplementary Material; further inquiries can be directed to the corresponding author.
