# Supplementary material for: Boosting the inherent activity of NiFe layered double hydroxide via erbium incorporation for water oxidation
Source: Front Chem. 2023 Aug 24;11:1261332. doi: 10.3389/fchem.2023.1261332 (PMC10483838; doi:10.3389/fchem.2023.1261332)
Supplement: Supplementary file 1 [file DataSheet1.pdf]

# *Supporting Information for*

# Boosting the Inherent Activity of NiFe Layered Double Hydroxide *via* Erbium Incorporation for Water Oxidation

*Jitao Yang* [§], *Yibin Yang* [*,§]

[§]School of Chemical and Pharmaceutical Engineering, Chongqing Industry Polytechnic College, Chongqing, 401120, P. R. China

**Corresponding Authors**

*E-mail: yangyb@cqipc.edu.cn (Yibin Yang);

# Additional Data and Figures

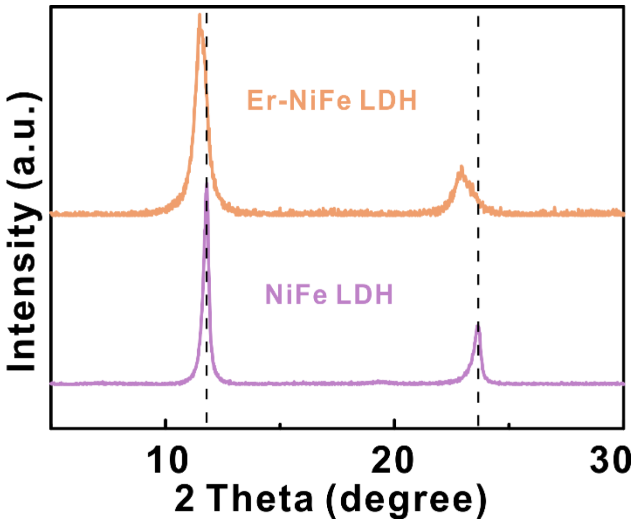


**Figure S1.** Partial enlarged PXRD pattern of Er-NiFe LDH and undoped NiFe LDH powders.

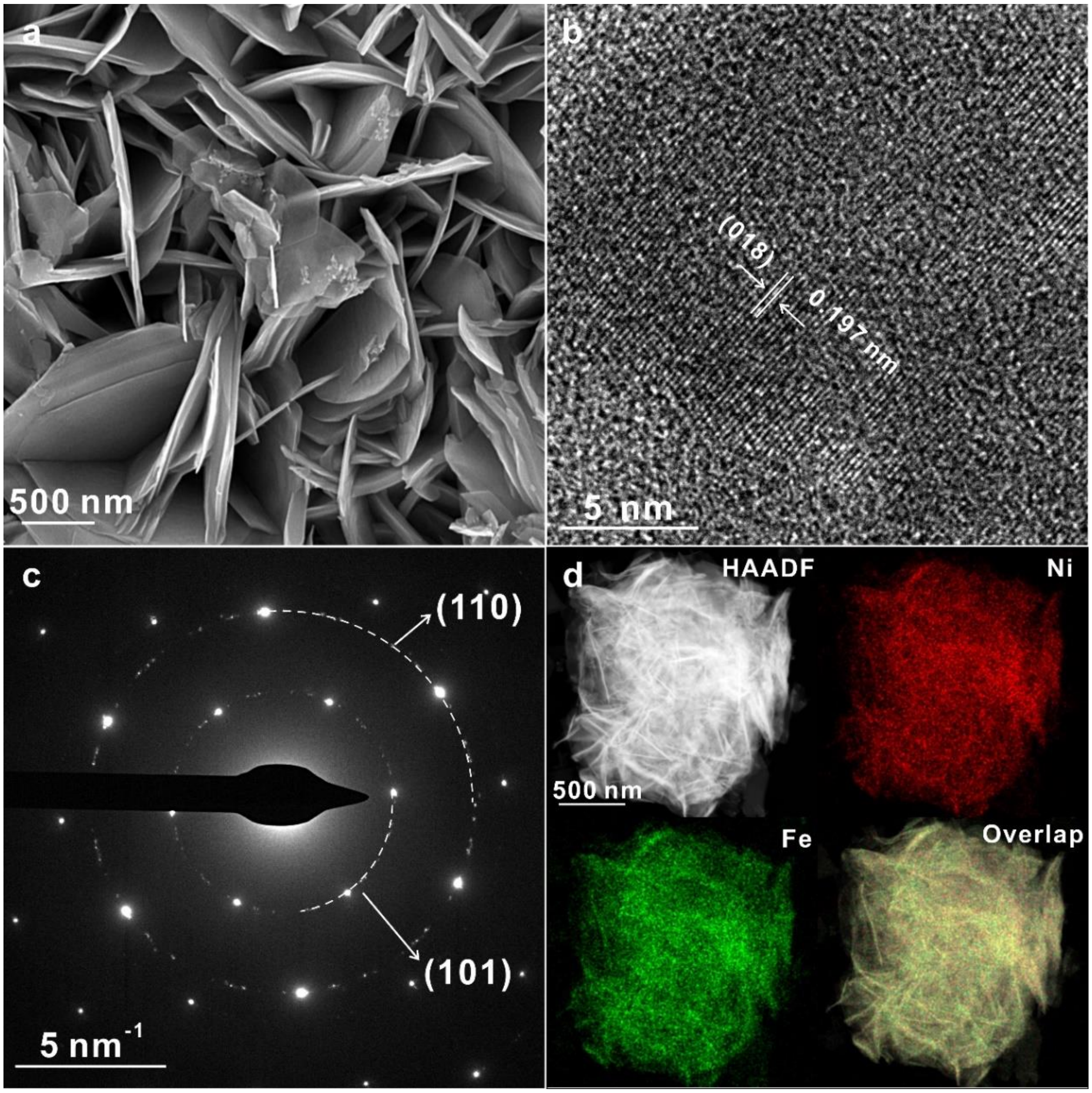


**Figure S2.** Material properties of NiFe LDH powder. (a) SEM image. (b) High-resolution TEM image. (c) SAED pattern. (d) HRTEM image and corresponding element distribution maps.

SEM image (Figure S1a) clearly shows the well-defined nanosheets structure of NiFe LDH powder. HRTEM image (Figure S1b) indicates the lattice stripe of 0.197 nm, which corresponds to the (018) plane of NiFe LDH. SAED pattern (Figure S1c) exhibits two distinct diffraction rings (101) and (110), which are matched well with the PXRD

results. Furthermore, HAADF-STEM and elemental mappings reveals that Ni and Fe elements are evenly distributed on the surface of NiFe LDH powder. These results suggests that the NiFe LDH powder was successfully synthesized *via* a facile hydrothermal reaction.

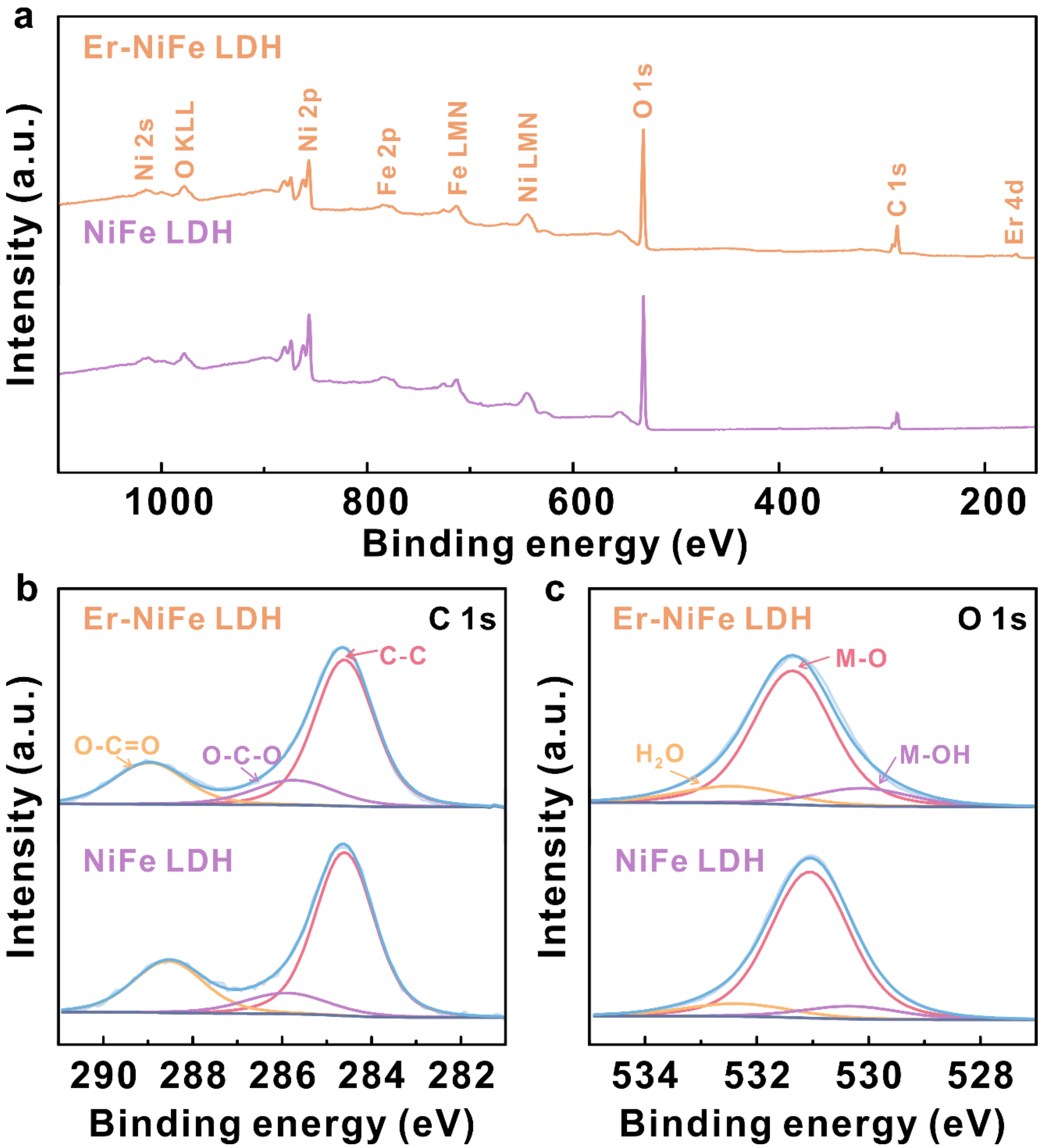


**Figure S3.** XPS spectra of (a) Survey, (b) C 1s, and (c) O 1s of Er-NiFe LDH and NiFe LDH powders.

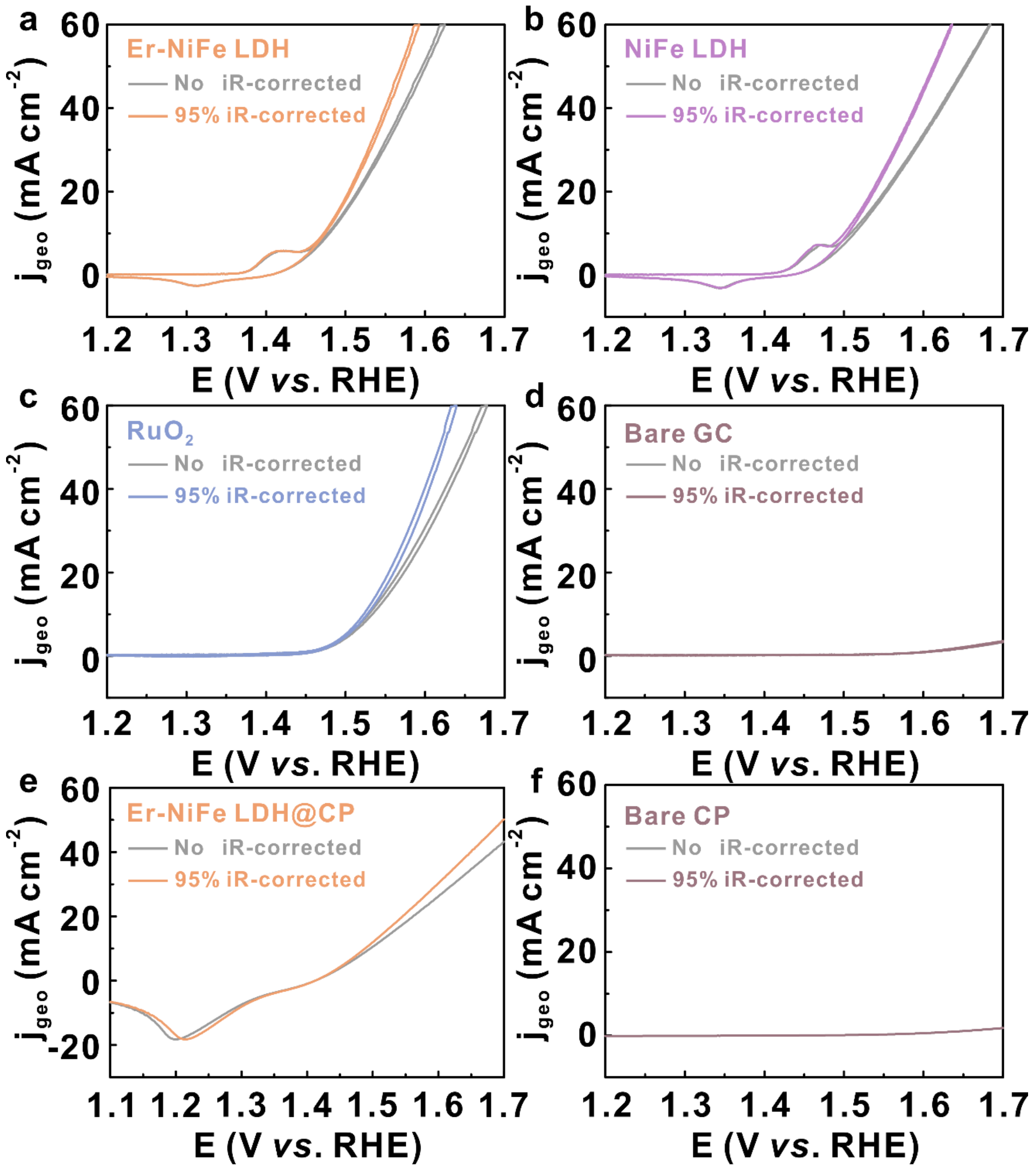


**Figure S4.** Polarization curves before and after 95% *i*R correction of the resistance of all LDH electrodes in 1.0 M KOH solution.

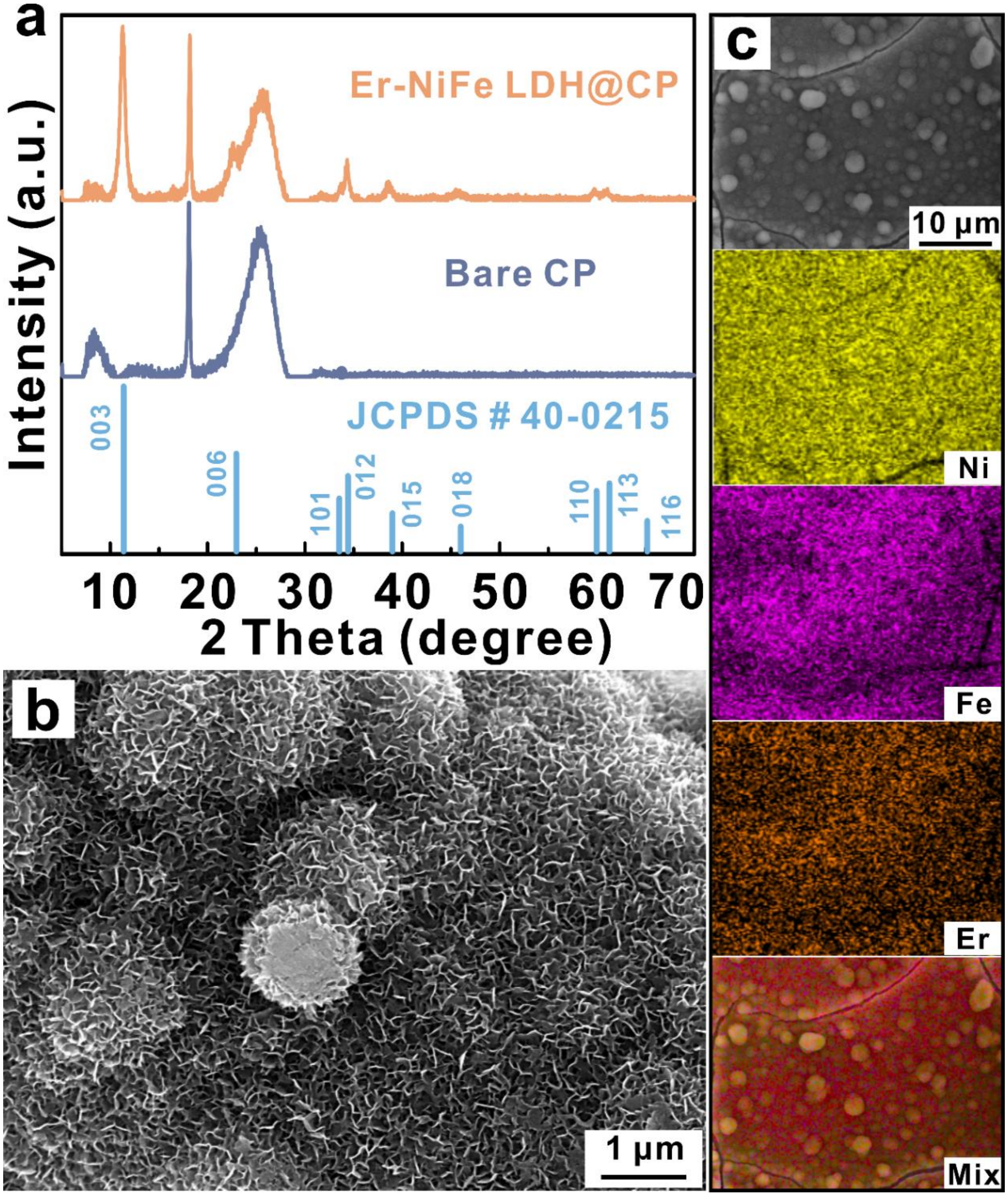


**Figure S5.** Material properties of Er-NiFe LDH@CP electrodes. (a) XRD patterns, (b) SEM image, and (c) EDS elemental maps.

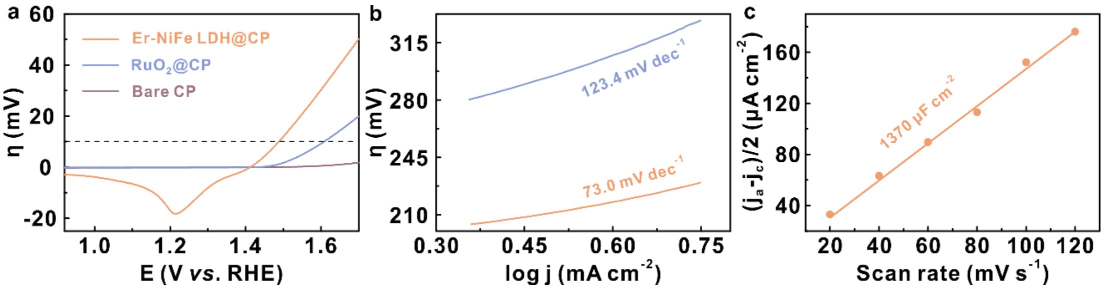


**Figure S6.** OER performance of 3D electrodes. (a) CV curves, (b) Tafel slope, and (c) $C_{dl}$.

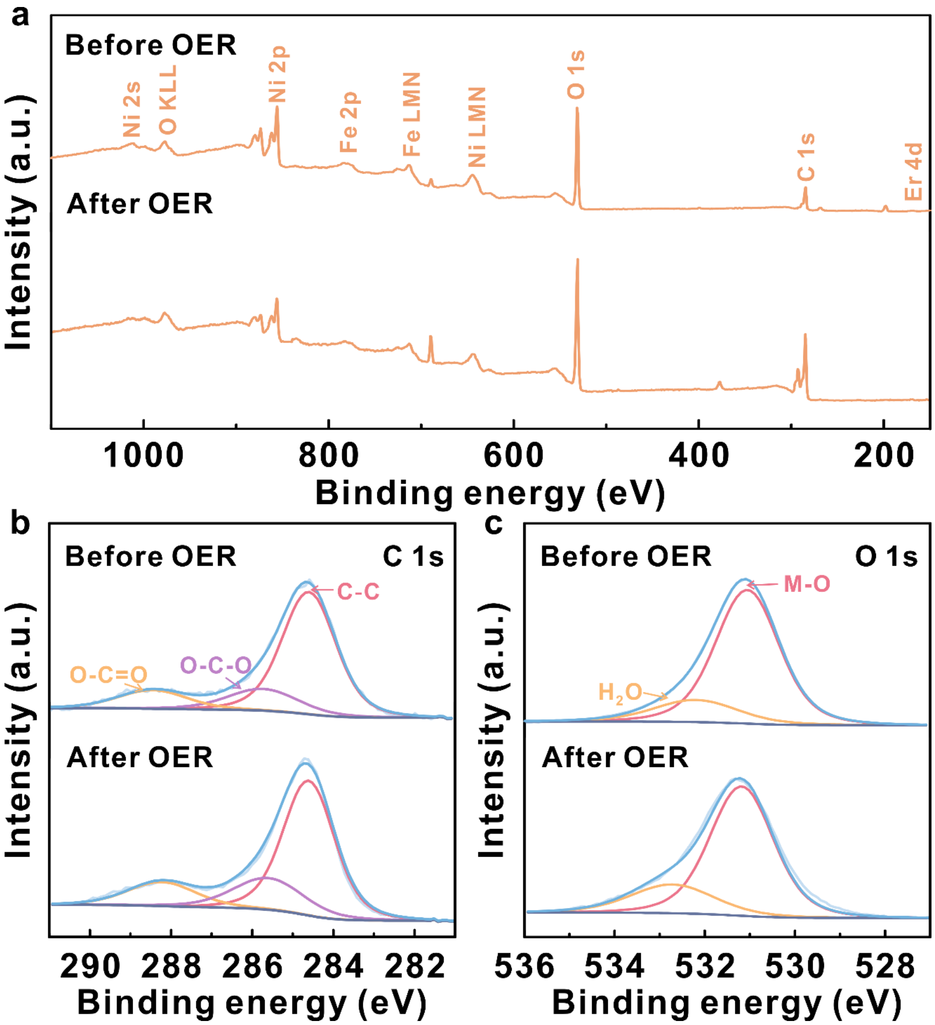


**Figure S7.** XPS spectra of (a) Survey, (b) C 1s, and (c) O 1s of Er-NiFe LDH@CP electrode before and after stability test.
